# Supplementary material for: Free Methylglyoxal as a Metabolic New Biomarker of Tumor Cell Proliferation in Cancers
Source: Cancers (Basel). 2024 Nov 22;16(23):3922. doi: 10.3390/cancers16233922 (PMC11640185; doi:10.3390/cancers16233922)
Supplement: Supplementary file 1 [file cancers-16-03922-s001.zip › cancers-3293593-supplementary.pdf]

cancers MDPI

Supplementary method

S1. Chemicals and technical devices used for methylglyoxal (MG) measurement

All chemicals were analytical grade. Methylglyoxal (MG) (40% aqueous solution), Sodium azide and DETAPAC was supplied by Sigma-Aldrich. O-phenylenediamine also called 1,2-diaminobenzene (DB) was purchased from AcrosOrganics-Fisher Scientific and 5-methylquinoxaline (5-MQX) from Interchim. Acetonitrile and trifluoroacetic acid (TFA) for High performance liquid chromatography (HPLC) gradient grade were purchased from Carlo Erba and formic acid (99-100%, Normapur) from Grosseron. Water used in these experiments was purified using a Milli-Q Water Purification System (EMD Millipore Corp.). The reversed phase chromatography column (symmetry C18: 3.9x150 mm; 5 µm) and the Symmetry Sentry™ guard column (3.9x20 mm, 5 µm) were purchased from Waters (Milford).

S2. Preparation of blood samples

The whole blood was collected on heparin from patients and healthy controls by conventional means, and immediately kept on ice before being frozen at -80°C. The freezing time did not exceed 3 months. Defrosting was gentle on ethanol ice mixture.

100 µl of whole blood was diluted with 50 µl of TFA (50 µl) and mixed by vortexing. The mixture diluted and mixed by vortexing with 100 µl of Milli-Q water and internal standard (5-MQX, 7 picomoles). 175 µl of supernatant obtained by centrifugation (12,000 21 g, 10 min, 4°C) was supplemented with sodium azide (25 µl) and DB HCl-DETAPAC (50 µl). Each sample were incubated for 4 h in the dark at room temperature.

Derivatized samples were then transferred into an autosampler at 4°C for analyze. Triplicate free MG measures were done.

We used trifluoroacetic acid (TFA) instead of trichloroacetic acid (TCA) to precipitate proteins because TCA gave us interferences causing background noise.

We also determined the significant difference between the use of whole blood and plasma for the measurement of free MG in blood. Figure S1 shown the difference established in 18 healthy controls. We obtained 0.039 +/- 0.002 µM free MG measurement for plasma and 0.088 +/- 0.006 µM for its measurement for the whole blood (p=0.001). Since this difference was reproducible and in the same ratio between patients and healthy controls, we used this measurement in both patients and controls. Free MG recovered from the whole blood may reflect both free MG from plasma and from red blood cells due to a free MG release caused by frosting/defrosting. Whatever the origin of free MG, dosages were done in controls and patients with the same methodology.

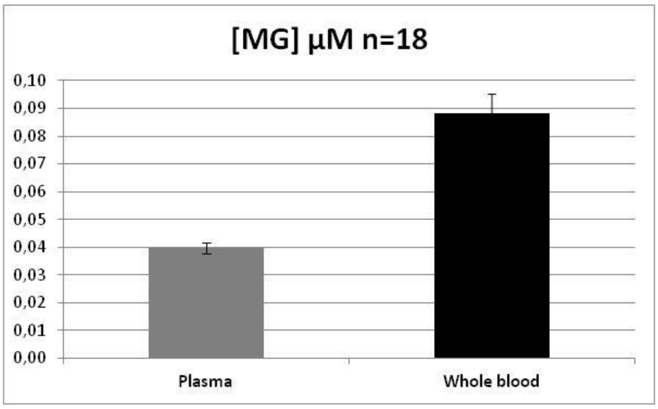

Figure S1. Free MG in plasma and whole blood.

S3. Preparation of tissue samples

The samples of tumors and of healthy tissues were done during surgery.

Tumoral and healthy tissue samples were cut with cryostat (15-20 thin sections of 60 µm). The thin sections were put in specific tubes at -20°C for sample preparation for Precellys homogenisers. 500 µl of water (4°C) were added. The mixture was homogenate with Precellys 2 x 5 sec with 10 sec break. 50 µl of the supernatant obtained by centrifugation 47 (12,000g , 10 sec, 4°C) was removed to determined protein concentration and the new mixture was diluted with 100 µl of TFA and mixed by vortexing. The supernatant obtained by centrifugation (20,000 g, 15 min, 4˚C) was supplemented with DB (20 µl). Each sample were incubated for 4 h in the dark at room temperature.

Derivatized samples were then transferred into an autosampler at 4 °C for analyze.

S4. Preparation of standards

Calibrating standards containing 0.0625-1.6 µmol of MG in 1 ml of water were prepared. Derivatization was carried out by the procedure described above. Calibration curves were constructed by plotting the pick area ratios of 2-methylquinoxaline (2-MQX) and 5-MQX internal standard against the MG concentrations.

S5. HPLC analysis

HPLC analysis of 2-MQX was performed on an LC-MS 8040 Shimadzu. The column is a Kinetex 2.6 µm EVO C10 100Å size 100 × 2.1 mm column. The sample temperature was maintained at 4 °C in the autosampler. The mobile phase A is 0.1% (vol/vol) Formic acid in water with a linear gradient of 0–100% solvent B over 10 min. The flow rate is 0.2 ml/min. The column is then washed for 5 min with 100% solvent B (0.1% formic acid 50% acetonitrile) and re-equilibrated for 15 min with 100% solvent A; the flow rate was increased to 0.4 ml/min for this stage.

S6. Mass spectrometric detection

Mass spectrometric detection 2-MQX was detected by electrospray positive-ion multiple reaction monitoring (MRM) with a retention time of 6.2 min. The injection volume was 10 µl. Two MRM mass transitions (molecular ion > fragment ion, Da) are recorded for analyte and internal standard. MRM mass transition, collision energy (eV) and cone voltage (V) are as follows: 2-MQX—145.1 > 77.1 and 145.1 > 92.1. Other mass spectrometer settings were as follows: capillary voltage, 0.60 kV; extractor voltage, 2.00 V; source temperature, 120 °C; desolvation gas temperature, 350 °C; desolvation gas flow, 900 liters per hour; and cone gas flow, 146 liters per hour.
